# Doctors' personal health care choices: A cross-sectional survey in a mixed public/private setting

**DOI:** 10.1186/1471-2458-8-183

**Published:** 2008-05-28

**Authors:** Julie Y Chen, Eileen YY Tse, Tai Pong Lam, Donald KT Li, David VK Chao, Chi Wai Kwan

**Affiliations:** 1 Family Medicine Unit, The University of Hong Kong, 3/F Ap Lei Chau Clinic, 161 Main Street, Ap Lei Chau, Hong Kong, PR China; 2 Department of Family Medicine and Primary Health Care, United Christian Hospital, G/F, Block G, 130 Hip Wo Street, Kwun Tong, Kowloon, Hong Kong, PR China; 3 Department of Statistics and Actuarial Science, The University of Hong Kong, Pokfulam Road, Pokfulam, Hong Kong, PR China

## Abstract

**Background:**

Among Western countries, it has been found that physicians tend to manage their own illnesses and tend not have their own independent family physicians. This is recognized as a significant issue for both physicians and, by extension, the patients under their care, resulting in initiatives seeking to address this. Physicians' personal health care practices in Asia have yet to be documented.

**Methods:**

An anonymous cross-sectional postal questionnaire survey was conducted in Hong Kong, China. All 9570 medical practitioners in Hong Kong registered with the Hong Kong Medical Council in 2003 were surveyed. Chi-square tests and logistic regression models were applied.

**Results:**

There were 4198 respondents to the survey; a response rate of 44%. Two-thirds of respondents took care of themselves when they were last ill, with 62% of these self-medicating with prescription medication. Physicians who were graduates of Hong Kong medical schools, those working in general practice and non-members of the Hong Kong College of Family Physicians were more likely to do so. Physician specialty was found to be the most influential reason in the choice of caregiver by those who had ever consulted another medical practitioner. Only 14% chose consultation with a FM/GP with younger physians and non-Hong Kong medical graduates having a higher likelihood of doing so. Seventy percent of all respondents believed that having their own personal physician was unnecessary.

**Conclusion:**

Similar to the practice of colleagues in other countries, a large proportion of Hong Kong physicians self-manage their illnesses, take self-obtained prescription drugs and believe they do not need a personal physician. Future strategies to benefit the medical care of Hong Kong physicians will have to take these practices and beliefs into consideration.

## Background

Doctors as a group tend to take a self-reliant view when it comes to taking care of their own health [1-6]. In fact, "doctors appear to be reluctant patients who look after their health in a haphazard way through kerbside consultation, self medication and self referral to specialist services, often inappropriately [7]."

In recognition of this pattern, many professional medical organizations or health authorities around the world, including Canada [8], the UK [9], the USA [10], Australia [11] and Ireland [12], have initiated measures in their jurisdictions aimed at optimizing the health care of their own physicians. For instance, the Canadian Medical Association has established the "Centre for Physician Health and Well Being", the Irish College of General Practitioners has implemented the "Health in Practice Programme" and the British Medical Association has the "Doctors for Doctors Unit". A variety of services are available including a listing of doctors who specialize in having doctors as patients, psychological services, programmes targeting trainees and students, and referral information. An international conference on physician health [13] now held annually lends credence to the growing significance and concern about this issue.

Acknowledgement has also reached government levels with a White Paper being published in the UK addressing the issue of ill health in health professionals. It states that "further measures should be put in place to provide effective arrangements to support health professionals in maintaining their own health... [9]." After all, unhealthy doctors cannot be expected to provide quality health care to their patients.

According to its most recent statistics, the World Health Organization estimated that there were approximately 2 449 036 physicians in Asia [14]. Not much, however, is known about what Asian physicians do in terms of their own health care. This present study aims to obtain preliminary information about this population by focusing on doctors in Hong Kong to find out what they do when they are ill, how they feel about having their own doctors and to identify the significant background characteristics of these physicians.

## Methods

A pilot questionnaire was first sent to a random sample of physicians and the resultant final version of the survey is appended (Additional file 1). This consisted of 23 items which asked about the use of medical services and medications when last ill, demographic information as well as specialty, educational background and clinical experience.

The questionnaire survey was sent to all 9570 local Hong Kong doctors registered with the Medical Council of Hong Kong in 2003. Three rounds of mailings were done between May and July 2003.

Ethics approval for this study was obtained from the Ethics Committee of the Faculty of Medicine, the University of Hong Kong.

The data were analyzed using descriptive statistics and comparison of proportions. The primary outcome measures were (a) whether respondents consulted another doctor when he or she was last ill and (b) whether they felt a personal physician was necessary for their own medical care, which were then cross-tabulated with independent background variables. The chi-square test was employed to test for the significance of the difference between group proportions. Logistic regression was applied to study the adjusted association of the background characteristics and outcomes. The statistically significant level was 5%. SAS 8.0 (Statistical Analysis Software, version 8.0) and SPSS 15.0 (Statistical Package for the Social Sciences software, version 15.0) were used for data analysis.

## Results

### Demographic data

4198 doctors responded to the survey (a 44.4% response rate). Three-quarters of respondents were male and obtained their medical degree in Hong Kong. Most were aged between 30 and 50. More than half were in public practice and one-third considered their main area of practice to be family medicine/general practice (FM/GP).

By way of background, in Hong Kong, all registered doctors are permitted to engage in general practice regardless of their specialist certification status. These include doctors who have not undergone further vocational training, specialists in family medicine/general practice who have undergone further training in family medicine, specialists in internal medicine or surgery or other areas of clinical medicine as well as specialists typically not involved in direct patient care such as pathology and community medicine.

The demographic breakdown of the respondents was generally consistent with the findings in the 2003 Health Manpower Survey [15] conducted on all local practising physicians by the Hong Kong Department of Health. Relevant information in the government survey included gender, age, type of practice, practice setting and specialty.

### What did doctors do when they were last ill, either physically or emotionally?

The survey indicated that the majority of respondents, 64% (N = 2701) did not consult another physician. Instead, they self-managed their illness, with 88% (N = 2357) of non-consulters taking some medications, 10% (N = 280) doing nothing and 2% (N = 50) doing other things like investigations, seeking physiotherapy or consulting alternative health practitioners.

Of those who took some medication, 62% (N = 1675) took prescription-only medication which was self-obtained.

The remaining one-third of respondents sought care from another physician.

### What were the characteristics of respondents who chose to consult another doctor when they were last ill?

Table 1 shows the distribution and statistically significant characteristics of physician respondents who consulted another medical practitioner. Age was found to be the main factor affecting whether a doctor was consulted, and age was related to all other background information. Therefore, the adjusted results differ from the general results due to the significance of age.

**Table 1:** Respondents who consulted another doctor when last ill

Variables*	Adjusted results				
	N	%	OR	(95% CI)	p-value
**Gender**					0.001
Male	1088	73	1		
Female	402	27	1.32	1.12–1.55	
					
**Age**					<0.001
20–29	159	11	1		
30–39	394	26	1.03	0.80–1.32	
40–49	327	22	1.03	0.72–1.48	
50–59	323	22	1.66	1.02–2.70	
60–69	195	13	2.40	1.27–4.53	
70+	91	6	4.46	1.94–10.27	
					
**Specialty**					<0.001
Surgeons	356	24	1.53	1.21–1.92	
Physicians	434	29	1.65	1.31–2.07	
FM/GP	463	31	1		
Others	231	15	1.71	1.31–2.23	
					
**HKCFP member**					<0.001
Yes	313	21	1		
No	1176	79	0.69	0.56–0.85	

*Medical degree, type of practice and practice setting were not statistically significant.

It was found that male doctors, doctors who earned their medical degrees outside of Hong Kong, FM/GP, non-members of the HKCFP, private practitioners and solo practitioners were older. It would thus be expected that all these doctors, because they were older, would be more likely to consult another physician when ill. However, after adjusting for age, only the characteristics of gender, specialty and HKCFP membership emerged as significant. Therefore, the general association between these characteristics and whether a doctor is consulted was compensated by the age effect. The statistically significant general association of where the degree was obtained (p < 0.001), the type of practice (p < 0.001) and the practice setting (p < 0.001) was actually a reflection of the significance of age.

### Which types of doctors were consulted by these respondents?

As seen in Table 2, 86% of all consultations were with health care providers other than FM/GP. Of these, the majority were physician specialists and general surgeons/other surgical specialists. Family medicine physicians or general practitioners were consulted by only 14% of respondents.

**Table 2:** Specialty of doctor consulted when last ill

Specialty	Frequency	Valid percent
Internal medicine	426	28.8
FM/GP	211	14.3
Surgery	208	14.1
Orthopaedics	140	9.5
Ophthalmology	136	9.2
ENT	107	7.2
Other specialties*	205	13.8
Other (Chinese medicine, etc.)	45	3.0
Total	1478	100.0

Note: 15 (1%) respondents did not answer

* including Paediatrics, O&G, Psychiatry, A&E, Radiology, Pathology, Community Medicine, Anaesthesiology.

After the adjustment of confounding variables, only age and medical degree were found to be significant, as shown in Table 3. The younger age group (ages 20–29) and holders of non-Hong Kong medical degrees were more likely to consult a FM/GP.

**Table 3:** Respondents who consulted a Family Medicine/General Practitioner when last ill.

Variables*	Adjusted results				
	N	%	OR	(95% CI)	p-value
**Age**					<0.001
20–29	64	30	1		
30–39	72	34	0.30	0.19–0.47	
40–49	37	18	0.17	0.10–0.30	
50–59	18	9	0.08	0.04–0.17	
60–69	15	7	0.12	0.05–0.25	
70+	4	2	0.07	0.02–0.24	
					
**Medical degree**					<0.001
Hong Kong	132	63	1		
Mainland China/Taiwan	19	9	2.09	1.09–3.99	
North America	2	1	1.57	0.34–7.31	
Australia	27	13	3.10	1.77–5.45	
Europe	24	11	2.42	1.36–4.29	
Others	8	4	2.10	0.81–5.42	

* Gender, type of practice, practice setting, specialty, HKCFP membership status were not statistically significant

The most important consideration when choosing whom to see was consultant specialty. This was mentioned by almost half of the physicians (48%, N = 1850) who had indicated that they had ever consulted another medical practitioner professionally (92% of all respondents, N = 3850). One-quarter of these physicians stated consultant experience as the most important factor and 18% indicated that the colleague be in the same institute or practice as the most important factor. The remainder said nothing in particular was taken into consideration.

### What were the characteristics of doctors who self-prescribed when last ill

After comparison of proportions and multiple logistic regression analyses as before, medical degree (p = 0.007), area of practice (p = 0.034) and HKCFP membership (p = 0.047) were significant. Compared with Hong Kong medical graduates, doctors who earned their medical degrees elsewhere were less likely to self-prescribe. North American medical school graduates were least likely to self-prescribe (OR 0.30, 95%CI 0.14–0.67). Compared with those engaged in family medicine/general practice, doctors who described their predominant area of practice as a medical specialty or a surgical specialty were less likely to self-prescribe than FM/GP (OR 0.89, 95%CI 0.67–1.18 and OR 0.71, 95%CI 0.53–0.96 respectively). Non-members of the HKCFP were more likely to self-prescribe (OR 1.29, 95%CI 1.00–1.67).

### Did doctors think they needed a personal physician?

Overall, less than one-third of all respondents (31%, 1287/4185) felt they needed a personal physician. Of those who self-prescribed for their last illness only 22% (364/1675) felt they needed a personal physician, while of those who consulted another doctor for their last illness 44% (654/1487), felt they did.

Table 4 shows the distribution of respondents who felt they needed a personal physician. The adjusted results showed that doctors who felt they needed a personal physician were more likely to be female, older than age 50, non-Hong Kong/Chinese medical degree holders, working in a community group practice and a member of the HKCFP.

**Table 4:** Respondents who believed they needed a personal physician

Variables*	Adjusted results				
	N	%	OR	(95% CI)	p-value
**Gender**					0.017
Male	928	72	1		
Female	360	28	1.23	1.04–1.45	
					
**Age**					<0.001
20–29	183	14	1		
30–39	339	26	0.78	0.60–1.00	
40–49	291	23	0.94	0.66–1.35	
50–59	261	20	1.38	0.85–2.27	
60–69	157	12	1.89	0.99–3.60	
70+	55	4	1.81	0.80–4.12	
					
**Medical degree**					<0.001
HK	807	63	1		
Mainland China/Taiwan	102	8	1.04	0.77–1.40	
North America	35	3	3.67	2.04–6.60	
Australia	129	10	2.34	1.78–3.06	
Europe	143	11	1.92	1.49–2.48	
Others	67	5	2.27	1.50–3.43	
					
**Practice setting**					0.018
Solo	465	38	1		
Group, community	265	22	1.18	0.92–1.52	
Group, hospital	480	40	0.84	0.62–1.15	
					
**HKCFP member**					<0.001
Yes	366	28	1		
No	916	71	0.54	0.44–0.66	

* Type of practice and specialty were not statistically significant

## Discussion

### Principal findings

It was found that a majority of physicians self-treated when last ill and did so with prescription medication. Most physicians did not believe they needed a personal physician to provide health care. For those who sought care from another physician, the specialty of the physician consulted was the most important factor in their decision of whom to consult. Only 14% consulted a FM/GP. These were more likely to be younger doctors and holders of medical degrees from non-Hong Kong universities. Self-prescribing doctors were more likely to be Hong Kong-educated, engaged in general practice and non-members of the HKCFP.

### Strengths and weaknesses

#### Study population

This is the first study to include all physicians listed on the medical register of a population. Previously, participants have been randomly sampled physicians or subgroups of physicians, such as particular specialties e.g. family physicians [4] or neurologists [16], or those under a certain age [17]. This is also the first study on doctors' illness behaviour in Asia.

The response rate was 44% which was in the low range when compared with studies by Pullen (44%, N = 1125)[3], Wachtel (67%, N = 306)[5], and Toyry (74%, N = 3313)[17]. In the context of Hong Kong based surveys of medical doctors, in which Leung and colleagues [18] concluded that the response rate is low (less than 20%) even when cash incentives are offered to respondents, this survey demonstrated a significantly higher response rate. Nonetheless, a low response rate undoubtedly limits the interpretation of the data.

When determining how representative our sample population was in relation to the general physician population, it was very noteworthy that there was no database of basic physician demographics to which we could compare our sample. As the Hong Kong Department of Health Manpower Survey [15] was the only information available, it was used as the basis for comparison, despite being a voluntary survey itself, with a 53% (N = 5276) response rate. Unknown response and selection biases also make it impossible to determine if this was a representative sample.

#### Rationale for the wording "last illness"

The choice of wording used, asking respondents to recall when they were "last ill", was deliberately set in order to tap individuals' subjective perception of being ill. By leaving it undefined we had hoped to include as wide an interpretation as possible which would encourage respondent consideration of mental and emotional conditions when answering the question.

Using broad, undifferentiated wording does prevent us from specifically assessing the "correctness" of the respondent's management decision. Even among doctors who sought medical attention the last time they were ill, we are unable to judge whether this was an "appropriate" consultation. We can only observe that the patient's perceived illness was such that they felt they needed to see a doctor. Similarly, for physicians who chose to self-medicate when they were last ill, their perception of being ill did not warrant seeing a doctor yet a majority considered themselves ill enough to take prescription medication.

Though not knowing the specifics and circumstances of the illness limits our judgement of the action, our data still enable us to determine that the practice of self-prescribing medication is common and that one-third of all respondents considered their last illness significant enough to seek medical attention.

The timing of the last illness was not specified with the aim of prompting respondents to recall an actual event. This allowed for information to be obtained from a different perspective than surveys which asked hypothetical questions or provided case scenarios in which the respondents would anticipate their actions in a given situation. However, the limitation is that answers would be dependent on respondent memory and truthfulness.

### Comparison with international findings and implications

#### Self-management

From the number of physicians who admitted to taking self-obtained prescription medication for their last illness in this survey, it would appear that a good proportion of these had "illness" that for lay people would require a visit to the doctor.

Doctors, however, clearly are a different population from the general public but they still get ill and perception of what constitutes "being ill" varies widely from individual to individual, as in lay people. Being trained doctors, with the knowledge and having the resources to take care of themselves, physicians can and do self-diagnose and self-treat when "ill" to a greater extent [17,19] with wide ranging opinions as to where the threshold for seeking help is. An Australian survey found that many physicians were prepared to treat themselves for serious illnesses [3]. Another showed that most doctors believed it was acceptable to self-treat for minor illness but were split over the self-management of chronic disease [2]. It has been shown for some chronic illnesses, self-management by patients, in collaboration with their doctor, can improve health outcomes [20] so there may be justification for physicians with chronic diseases to do the same.

Statements from the General Medical Council and Australian Medical Association [21,22] have also noted that it "makes sense to treat minor ailments" and emergencies. However, the main theme reiterated by medical associations and colleges is the concern about compromising clinical judgement and objectivity when the physician is treating himself. This has resulted in the recommendation discouraging physicians from self-treating in most circumstances [21,23,24].

The Medical Council of Hong Kong does not specifically address this issue although it implies a similar viewpoint as it stated with reference to issuing sick leave certificates, "...a doctor cannot be his own patient..." [25]. The Hong Kong Medical Association Ethics Committee indicated that there was, "...no government regulation nor any specific guidance in the Professional Code and Conduct, on the treatment of one's own/family members' illnesses although doctors should be cautioned against doing so..." in a written personal communication.

Despite the well-meaning aim of promoting prudent self-care practices among physicians and protecting patients, these ethical guidelines are not necessarily followed [26]. On the other hand, the effectiveness of the more forceful option of governmental legislation (against self-prescribing) on physician impairment is uncertain with a paucity of research noted in this area [27]. This issue of physician self-prescribing is summed up in a telling comment from Rosvold and Tyssen [28], "self-prescribing is not to be viewed simply as a cause of physicians' impairment, but more of a symptom of poor health-care for physicians".

Even among those who had ever seen a doctor before, the majority indicated that the most important reason for choosing the doctor that they did was physician specialty. Of those who sought consultation for their last illness, only 14% chose to consult a FM/GP which suggested that there was a degree of self-diagnosis and self-referral occurring. Having an initial assessment by an objective primary care physician, preferably one's own, did not appear to be common practice. However, it must be recognized that in the Hong Kong setting, primary care medicine is also practised by community based specialists in the sense that these physicians may provide general outpatient care as well as specialist outpatient care. In this study, it was not possible to determine whether the specialty certification status of the doctor consulted matched his/her predominant area of practice.

#### Personal physician

One of the pillars of recognized good health care for any individual is having a designated primary care physician to provide and coordinate care and is recommended in policy or position statements of respected medical associations [22,23]. It has been shown that there is an association between doctors who have a family physician and compliance with preventive health behaviour [29,30] and that these doctors are three times more likely to visit a physician for health maintenance than those without a family physician [31]. "Doctors themselves are concerned about the current level of illness within the profession and securing appropriate personal health care might be regarded as essential" [1].

Despite this, only 30% of respondents felt they needed a personal physician. This compares unfavourably with surveys of physicians in various other countries. In an Australian survey, 42% had a GP although less than a third actually consulted their GP for health problems [3]. In Rhode Island, USA two-thirds of physicians indicated that they had a primary care physician [5] and in New Zealand, 71% claimed to have one [6]. This discrepancy may be due in part to the Hong Kong health care system in which a solid primary care infrastructure is still lacking, when compared to these more established systems. As well, our survey did not measure the numbers of respondents who actually had a personal physician, only their perceived need for one so this 30% may, in fact, be an overestimation.

Being female and having membership in the HKCFP were found to be related to the generally more healthy practices of consulting another doctor when ill and believing in having a personal physician. Female doctors in Australia have also been shown to be more amenable to seeing another physician for medical care and were more likely to discuss problems with their doctor [3]. Presumably members of the HKCFP would have a greater interest, and vested interest, in promoting and propagating the concept of quality primary care. Many may have pursued further training, and thus have gained a better understanding of the principles of family medicine, including the health benefits of having a personal primary care physician.

## Conclusion

This study provides some preliminary information about the health care practices of physicians in Hong Kong. The findings that the majority of physicians self-treat, with a large proportion self-prescribing, and don't believe in having a personal physician suggest that this is a population with personal health care practices which warrant a closer look.

It would be worthwhile to document the actual state of doctors' health via a morbidity survey linked to personal health care practices. Investigating the perceived barriers to obtaining medical care by doctors in Hong Kong and qualitative studies on why doctors self-medicate and don't believe in having a personal physician would add further useful information in the assessment of the state of doctors' health in Hong Kong. Such issues have been examined in other countries notably the UK [1] and Australia [2,32] and although the conclusions may be extrapolated to some extent, specific local data would be of value in determining the need and/or focus of any doctor-specific health care initiatives in Hong Kong.

## Competing interests

The authors declare that they have no competing interests.

## Authors' contributions

JYC was involved in reviewing/analyzing the data and drafted the manuscript. EYYT was the principal investigator of the study who was involved in initiating/designing the study and acquiring/analyzing the data. TPL was involved in initiating/designing the study and supervised the data collection/analysis. DKTL and DVKC contributed to the design of the study and data analysis. CWK did the statistical analyses and contributed to the interpretation of data. All authors read and approved the final manuscript.

## Pre-publication history

The pre-publication history for this paper can be accessed here:



## Supplementary Material

Additional file 1 **Appendix 1:** Questionnaire Survey. Click here for file
