# Supplementary material for: Doctors' personal health care choices: A cross-sectional survey in a mixed public/private setting
Source: BMC Public Health. 2008 May 28;8:183. doi: 10.1186/1471-2458-8-183 (PMC2429910; doi:10.1186/1471-2458-8-183)
Supplement: Additional file 1 — Appendix 1: Questionnaire Survey. [file 1471-2458-8-183-S1.DOC]

**Appendix 1** – Questionnaire Survey

SURVEY ON THE USE OF MEDICAL CARE BY DOCTORS AND THEIR FAMILIES

1. Did you consult a doctor when you were last ill (physically or emotionally)? ⬜

1 = Yes 2 = No

1. If yes, what was the specialty of the doctor? (Please choose ONE only) ⬜ ⬜

01 = Internal Medicine 02 = Surgery

03 = O&G 04 = Paediatrics

05 = Orthopaedics 06 = Psychiatry

07 = A&E 08 = Radiology

09 = Pathology 10 = ENT

11 = Community Medicine 12 = Anaesthesiology

13 = Ophthalmology 14 = Family Medicine / General Practice

15 = others Please specify_________________________

1. If not, what did you do? ⬜

1 = Nothing 2 = Took some medication 3 = others Please specify____________

1. If you took some medication, was it ⬜

1 = over the counter medications 2 = prescription only medications 3 = both

1. When did you last consult a medical practitioner professionally? ⬜

1 = never (Go to Question 8 if your answer is never)

2 = within 6 months

3 = within one year

4 = within five years

5 = more than five years

1. If ever, were you admitted to hospital for treatment at this last consultation? ⬜

1 = Yes 2 = No

1. If ever, how did you decide on whom to choose? (Please take only

one category which is the most important consideration.) ⬜

1 = a colleague in the same institute / practice 3 = his/her clinical experience

2 = his/her specialty 4 = doesn’t matter

1. When was the last time you self-medicated? ⬜

1 = within 7 days 3 = more than one month but less than 3 months

2 = 7 to 30 days 4 = more than 3 months ago

1. What did you take? (Can choose more than one) ⬜ ⬜

01 = antibiotics

02 = analgesics

03 = drugs for URTI

04 = drugs for respiratory system (excluding URTI)

05 = drugs for gastrointestinal system

06 = drug for endocrine system (including contraceptives and hormonal replacement therapy)

07 = psychotropic drugs

08 = drugs for chronic illnesses (e.g. diabetes, hypertension or asthma)

09 = others Please specify _________________________

10 = combination Please specify _________________________

1. Do you think you need a personal physician? ⬜

1 = Yes 2 = No

1. Do you think your family members need a personal physician? ⬜

1 = Yes 2 = No

1. The last time a member of your immediate family was ill, who looked after him/her? ⬜

1 = by own self 2 = another doctor

1. Who normally looks after the health problems of your family members? ⬜

1 = by own self 2 = another doctor

1. If by another doctor, is he/she always the same one? ⬜

1 = Yes 2 = No

1. If yes, what is his/her specialty? (Please choose ONE only) ⬜ ⬜

01 = Internal Medicine 02 = Surgery

03 = O&G 04 = Paediatrics

05 = Orthopaedics 06 = Psychiatry

07 = A&E 08 = Radiology

09 = Pathology 10 = ENT

11 = Community Medicine 12 = Anaesthesiology

13 = Ophthalmology 14 = Family Medicine / General Practice

15 = others Please specify _________________________

1. What is your sex? ⬜

1 = male 2 = female

1. What was your age at last birthday? ⬜

1 = 20 to 29 4 = 50 to 59

2 = 30 to 39 5 = 60 to 69

3 = 40 to 49 6 = 70 or above

1. Where did you obtain your basic medical degree? ⬜

1 = Hong Kong 4 = Australasia

2 = Mainland China or Taiwan 5 = Europe (including UK & Ireland)

3 = North America 6 = others Please specify _______________

1. YEARS of clinical practice since graduation. ⬜ ⬜

1. What is the type of your practice? ⬜

1 = private 2 = public

1. Is your practice a group or solo practice? ⬜

1 = solo 2 = group, community based 3 = group, hospital based

1. What is your specialty? ⬜ ⬜

(If more than one, please select the one which represents most of your daily practice)

01 = Internal Medicine 02 = Surgery

03 = O&G 04 = Paediatrics

05 = Orthopaedics 06 = Psychiatry

07 = A&E 08 = Radiology

09 = Pathology 10 = ENT

11 = Community Medicine 12 = Anaesthesiology

13 = Ophthalmology 14 = Family Medicine / General Practice

15 = others Please specify _________________________

1. Are you a member of the Hong Kong College of Family Physicians? ⬜

1 = Yes 2 = No
